# Ectopic Prostatic Tissue in the Uterine Cervix Following Vaginal Hysterectomy

**DOI:** 10.7759/cureus.78557

**Published:** 2025-02-05

**Authors:** Abayomi I Alao, Nirav Gandhi, Bhawana Purwar

**Affiliations:** 1 Obstetrics and Gynaecology, University Hospitals of Derby and Burton NHS Foundation Trust, Derby, GBR; 2 Pathology, University Hospitals of Derby and Burton NHS Foundation Trust, Derby, GBR; 3 Urogynaecology, University Hospitals of Derby and Burton NHS Foundation Trust, Derby, GBR

**Keywords:** cervix uteri, ectopic tissue, hysterectomy, prostate, vaginal

## Abstract

Ectopic prostatic tissue (EPT) in the female genital tract is a relatively rare histopathological finding. EPT occurs when tissue from one region is found in another region, a phenomenon observed in various medical cases. However, the presence of ectopic tissue specific to one gender in another is uncommon.

An 83-year-old woman presented to the gynaecology outpatient clinic with symptoms of uterovaginal prolapse and subsequently underwent a vaginal hysterectomy. Histologic examination of the uterus revealed the presence of mixed glandular and squamous prostatic elements within the cervical tissue.

The exact embryological origins of cervical ectopic prostatic tissue remain incompletely understood. It is likely that a combination of genetic, hormonal, and developmental factors contributes to its occurrence.

## Introduction

Ectopic prostatic tissue (EPT) in the female genital tract is a relatively rare histopathological finding [1]. Ectopic tissue occurs when tissue from one region is found in another region, a phenomenon observed in various medical cases. However, the presence of ectopic tissue specific to one gender in another is uncommon [2].

In male embryonic development, the pelvic urethral endoderm gives rise to the prostate, whereas in females, it develops into the paraurethral Skene glands [2]. Reports have documented instances of EPT in female genital organs such as the ovary, cervix, and uterus. The underlying cause of this occurrence remains incompletely understood and is often an incidental histological finding [3].

Our report details a case in which ectopic prostatic tissue was incidentally discovered in the cervix following a vaginal hysterectomy.

## Case presentation

An 83-year-old para 2 woman presented to the gynaecology outpatient clinic with symptoms of uterovaginal prolapse. Despite attempting various sizes of ring pessaries, she experienced no substantial improvement. She did not report any lower urinary symptoms, bowel issues, or unusual vaginal discharge or bleeding. On examination, a stage 2 utero-vaginal prolapse was observed. She was otherwise fit and healthy.

She was counselled on management options and opted for a vaginal hysterectomy with pelvic floor repair. She subsequently underwent a vaginal hysterectomy and anterior repair, and the samples were sent for histology. She recovered well postoperatively and was discharged home on the first postoperative day.

**Figure 1:**
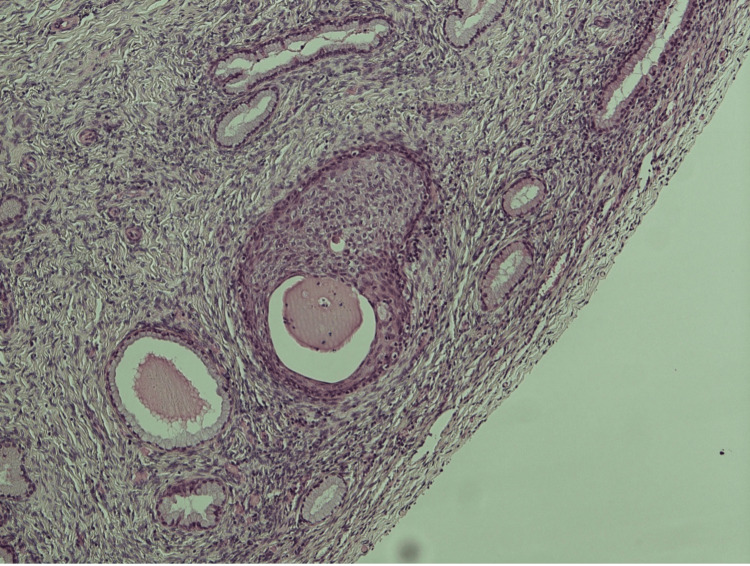
Photomicrograph (x100) of cervical tissue showing mixed glandular and squamous elements prostatic differentiation (PSA and NKX3.1 positive) with no evidence of malignancy. PSA: prostate-specific antigen stain.

On histology (Figure 1), the uterus and cervix measured 75 × 38 × 23 mm. On slicing the uterus and cervix, a polyp measuring 6 mm was noted at the fundus. Examination revealed the presence of mixed glandular and squamous prostatic elements within the cervical tissue. Histologically, the prostatic component displayed characteristic glandular structures with secretory acini and a distinct epithelial lining of prostatic tissue. Simultaneously, the squamous elements exhibited stratified squamous epithelium with keratinization. There was no evidence of CIN (cervical intraepithelial neoplasia), CGIN (cervical glandular intraepithelial neoplasia), or malignancy.

## Discussion

Ectopic prostatic tissue (EPT) refers to prostatic tissue that is located outside the prostate gland [4]. This condition was first reported by Jores in 1894 [1]. In men, a common type of non-cancerous EPT is the prostatic-type polyp, which is typically found near the prostate [5]. However, there have been rare cases of EPT found in women, primarily in the cervix, vagina, and ovaries. Unfortunately, these cases are not yet fully recognized by pathologists [1].

The embryological origins of cervical ectopic prostatic tissue remain a subject of ongoing research and speculation. Several theories have been proposed to explain the presence of prostatic tissue in the cervical region [5,6]. Some researchers suggest that abnormalities in the development of the Müllerian ducts, which give rise to the female reproductive tract, could lead to the development of ectopic prostatic tissue in the cervix. It is possible that remnants of male urogenital structures may persist in female embryos, leading to prostatic tissue development in unexpected locations [5]. Genetic factors and mutations may play a role in the development of cervical ectopic prostatic tissue. Certain genetic abnormalities could predispose individuals to the formation of prostatic tissue in unexpected locations [5]. Some experts propose that metaplastic changes in cervical epithelial cells could result in the transformation of these cells into prostatic tissue. This transformation could be driven by factors such as chronic inflammation or hormonal fluctuations [5-7]. The most widely accepted probability is that EPT in the cervix and vagina is probably a developmental anomaly. Cervical EPT may be derivatives of Skene’s gland, possibly homologous with the male prostate gland. Skene’s glands have similar embryogenic origins to the prostate gland, which is the urogenital sinus [8,9].

In 2014, the World Health Organization classified tumours of the female reproductive organs and briefly mentioned EPT. However, there is still a high rate of misdiagnosis due to inadequate knowledge [1]. Cervical EPT has been discovered incidentally in patients who have undergone loop electrosurgical excision or hysterectomy. It has been reported in various women [1]. This was also the case in the present patient, where we discovered cervical EPT following a vaginal hysterectomy.

EPT on histology mimics normal prostatic acini, typically with the epithelial cells on the luminal surface forming inverted papillae or cribriform structures [1]. According to reports, most cases of EPT in the genitourinary tract had lesions below the normal surface of the epithelium. In our specific case, upon examination, mixed glandular and squamous elements were found within the cervical tissue, but there was no sign of invasive growth or malignancy [1].

A possible differential diagnosis for cervical EPT is mesonephric remnants, which usually occur in the cervix and vaginal sidewalls. The ducts are small and relatively round with eosinophilic secretions within the cavity in EPT, while mesonephric remnants have a pathognomonic presence of a single layer of epithelium without peripheral basal cells [1]. Cervical EPT is a rare and often incidental finding, posing diagnostic challenges for pathologists [1]. Misdiagnosis is common due to its resemblance to other cervical lesions, such as mesonephric remnants. Cervical EPT is typically benign; however, its recognition is important to avoid misdiagnosis and unnecessary interventions [1].

## Conclusions

In conclusion, cervical ectopic prostatic tissue is a rare finding with unclear embryological origins. This case highlights the importance of histopathological awareness to ensure an accurate diagnosis. Further research is needed to elucidate the precise mechanisms underlying this rare phenomenon.
